# Pharmacogenetics of uridine diphosphate glucuronosyltransferase (UGT2B7) genetic polymorphism on valproic acid pharmacokinetics in epilepsy

**DOI:** 10.1186/1471-2164-15-S2-P3

**Published:** 2014-04-02

**Authors:** Murali Munisamy, Gauthaman Karunakaran, Mubarak Al-Gahtany, Vivekanandhan Subbiah, Manjari M Tripathi

**Affiliations:** 1 Department of Pharmaceutics, College of Pharmacy, King Khalid, University, Abha, Saudi Arabia; 2 Department of Pharmacology, College of Pharmacy, King Khalid University, Abha, Saudi Arabia; 3 Faculty of Neuro Surgery, King Khalid University, Abha, Saudi Arabia; 4 Neurosciences Centre, All India Institute of Medical Sciences, New Delhi, India

## Background

Sodium valproate is a widely prescribed broad-spectrum antiepileptic drug. It shows high inter-individual variability in pharmacokinetics and pharmacodynamics and has a narrow therapeutic range [1]. We evaluated the effects of polymorphic Uridine diphosphate glucuronosyltransferase (UGT2B7) metabolizing enzyme on the pharmacokinetics of sodium valproate in the patients with epilepsy who showed toxicity to therapy.

## Materials and methods

Genotype analysis of the patients was made with polymerase chain–restriction fragment length polymorphism (RFLP) with sequencing. Plasma drug concentrations were measured with reversed phase high-performance liquid chromatography (HPLC) and concentration–time data were analyzed by using a non-compartmental approach.

## Results

The results of this study suggested a significant genotypic as well as allelic association with valproic acid toxicity for UGT2B7 polymorphic enzymes. The elimination half-life (t_1/2_=42.2 h) of valproic acid was longer and the clearance rate (CL=947 ml/h) was lower in the poor metabolizers group of UGT2B7 polymorphism who showed toxicity than in the intermediate metabolizers group (t1/2 = 36.5 h, CL = 1,042 ml/h) or the extensive metabolizers group (t1/2 = 27. h, CL = 1,602 ml/h).

## Conclusions

Our findings suggest that the UGT2B7 genetic polymorphism plays a significant role in the steady state concentration of valproic acid, and it thereby has an impact on the toxicity of the valproic acid used in the patients with epilepsy.
